# Stem cell transplantation therapy in Parkinson’s disease

**DOI:** 10.1186/s40064-015-1400-1

**Published:** 2015-10-13

**Authors:** Mu-Hui Fu, Chia-Ling Li, Hsiu-Lien Lin, Pei-Chun Chen, Marcus J. Calkins, Yu-Fan Chang, Pei-Hsun Cheng, Shang-Hsun Yang

**Affiliations:** Institute of Basic Medical Sciences, College of Medicine, National Cheng Kung University, Tainan, 70101 Taiwan; Department of Physiology, College of Medicine, National Cheng Kung University, Tainan, 70101 Taiwan; Institute of Clinical Medicine, College of Medicine, National Cheng Kung University, Tainan, 70101 Taiwan; Department of Neurology, Kaohsiung Chang Gung Memorial Hospital and Chang Gung University College of Medicine, Kaohsiung, 83301 Taiwan; Division of Breeding and Genetics, Livestock Research Institute, Council of Agriculture, Tainan, 71246 Taiwan

**Keywords:** Stem cells, Cell replacement therapy, Neurodegenerative disease, Parkinson’s disease

## Abstract

Ineffective therapeutic treatments and inadequate repair ability in the central nervous system are disturbing problems for several neurological diseases. Fortunately, the development of clinically applicable populations of stem cells has provided an avenue to overcome the failure of endogenous repair systems and substitute new cells into the damaged brain. However, there are still several existing obstacles to translating into clinical application. Here we review the stem-cell based therapies for Parkinson’s disease and discuss the potential advantages and drawbacks. We hope this review may provide suggestions for viable strategies to overcome the current technical and biological issues associated with the application of stem cells in Parkinson’s disease.

## Background

Stem cells are undifferentiated cells that are able to differentiate into multiple specialized cell types. Since stem cells have the potential to replace or restore lost cells, they have been evaluated and considered as potential therapeutic agents in neuronal diseases. Numerous studies have focused on stem cell therapy in spinal cord injury, spinal muscular atrophy, brain ischemia, amyotrophic lateral sclerosis and other neurodegenerative diseases (Nicaise et al. 2015; Mendonca et al. 2015; Lukovic et al. 2015; Frattini et al. 2015; Ju et al. 2014). Because neurodegenerative diseases are often associated with regional cell loss, cell transplantation therapies may effectively restore and replace cells in the damaged tissues. Therefore, we will highlight several milestones in the development of stem cell therapy for Parkinson’s disease (PD), which is the second most common neurodegenerative diseases.

## Characteristics of various stem cells for therapy

There are several types of stem cells under consideration for therapeutic purposes. Below we will introduce four kinds of stem cells, including embryonic stem cells (ES cells), induced pluripotent stem cells (iPSCs), neural stem cells (NSCs) and mesenchymal stem cells (MSCs).

### Embryonic stem cells (ES cells)

ES cells are pluripotent cells derived from the inner cell mass (ICM) of blastocysts. These cells are able to differentiate into three germ layers, and subsequently may be driven to develop into many different types of cells (Thomson et al. 1998). In neuronal systems, prior studies have showed that functional neurons, astrocytes, and oligodendrocytes could be derived from ES cells in vitro (Wichterle et al. 2002; Zhang et al. 2001). As a result, ES cells transplant has been widely suggested in several neurodegenerative diseases or brain injuries (Aleynik et al. 2014). However, their high capacity of self-renewing and pluripotency lead to high risk of tumor formation, especially teratoma (Gordeeva 2011). Another major limitation is the ethical issue regarding their origin. Isolating ICM from blastocysts destroys early embryos and raises the moral concern (Daar and Sheremeta 2003). Due to the high tumorigenicity and ethical considerations, non-ES cells have become a major focus of cell-based therapies, such as adult stem cells.

### Induced pluripotent stem cells (iPSCs)

In 2006, Kazutoshi Takahashi and Shinya Yamanaka established the induced pluripotent stem cells, which are ES-like cells transformed from fibroblasts (Takahashi and Yamanaka 2006). This method is accomplished by introducing four transcription factor genes encoding Oct4, Sox2, Klf4, and c-Myc into skin fibroblasts. Since iPSCs may be derived directly from adult tissues, the risk of immune rejection and complicated ethical issues are avoided when used as a substrate for transplantation. Therefore, iPSCs were recently used as a potential cell source to repair neuronal networks in various CNS diseases, such as ischemic stroke and PD (Wernig et al. 2008; Yuan et al. 2013).

However, one major drawback of the iPSC technology is that c-Myc is well-defined as an oncogene, and reactivation of c-Myc increases the risk of tumor formation (Kawai et al. 2010). Yamanaka et al. modified the reprogramming protocol by using only Oct4, Sox2 and Klf4 without c-Myc, and it significantly decreased the tumorigenicity; however, this modified method significantly reduced the efficiency of iPSC formation (Nakagawa et al. 2008). Furthermore, Oct4, Sox2 and Klf4 are overexpressed or activated in various types of cancer as well (Peng et al. 2010; Raguel et al. 2009; Sholl et al. 2010), suggesting high risk of tumorigenicity as using these cells for transplantation. Recently, Chiou et al. (2013) reported that poly (ADP-ribose) polymerase 1 (Parp1) could be used for iPSC production, and it significantly decreases the risk of tumorigenicity, implying the major drawback could be overcome. However, the risk of teratoma formation after iPSCs transplantation could not be completely eliminated (Petit et al. 2014). Despite the obvious potential of iPSCs for cell-based therapy, this major hurdle should still be overcome before clinical use can be attempted.

### Neural stem cells (NSCs)

NSCs are stem-like progenitor cells that are isolated from either fetal brains or specific regions in adult brains (Kelly et al. 2004; Kukekov et al. 1999). In adult tissue, the subgranular zone (SGZ) in the dentate gyrus of the hippocampus and the subventricular zone (SVZ) of the lateral ventricles are two restricted regions producing NSCs, and these two regions confer neurogenesis in adult brain (Ming and Song 2011). NSCs are multipotent stem cells and recapitulate the developmental restriction toward a neural lineage. Therefore, these cells could be differentiated into neurons, astrocytes and oligodendrocytes (Jiang et al. 2012). Due to this specific lineage restriction, the risk of tumor formation is reduced, and NSCs are more easily guided toward neuronal differentiation. However, these cells cannot be isolated in large numbers, and it is also challenging to maintain or expand the cells in vitro over long periods of time (Anderson et al. 2007). As a result, the application of NSCs for transplantation is still limited.

### Mesenchymal stem cells (MSCs)

Mesenchymal stem cells are non-hematopoietic and multipotent cells first retrieved from the stromal area of the adult bone marrow (Fridenshtein 1991). In addition to bone marrow, MSCs may also be derived from a variety of non-marrow tissues, including placenta, muscle, skin, dental pulp, adipose tissue, umbilical cord and amniotic fluid (Jiang et al. 2012; Minguell et al. 2001). Since they can be retrieved from adult tissues, ethical concerns for MSCs could be avoided. Furthermore, one unique property of MSCs is immunomodulation, which may allow the cells to escape the surveillance of the host’s immune system or reduce the immune response of hosts (Guo et al. 2014; Glenn and Whartenby 2014). This characteristic would be an important concern for use in transplantation.

#### Bone marrow MSCs (BMSCs)

Bone marrow is the most common tissue from which MSCs are derived. The advantage of BMSCs is that these cells are relatively easy to be collected from patients’ own bone marrow without further CNS damage. Therefore, BMSCs may provide a relatively safe, ethical and immunologically favorable source for transplantation. In addition, application of BMSCs for the treatment of hematopoietic diseases began decades ago, which suggests that the protocol of isolation has been well established. As a result, BMSCs are considered as a resource with easier access. Another important feature is that BMSCs are able to cross the blood brain barrier and migrate throughout the brain (Li et al. 2001). This important advantage suggests the possibility that reconstruction/replacement of damaged brain tissues may be initiated via peripheral delivery without invasive methods. Furthermore, several reports have shown that BMSCs could be differentiated into neuronal cells (Zhao et al. 2015; Haragopal et al. 2015). These results support the therapeutic potential of BMSCs for neurological disease. However, the efficiency of differentiation into neuronal cells is low, and these cells may only be maintained for a few passages (Long et al. 2005). These drawbacks limit the potential application of BMSCs for transplantation.

#### Umbilical cord blood (UCB) cells

UCB is collected from the umbilical cord attached to the placenta during birth. UCB is comprised of hematopoietic stem cells, endothelial cell precursors, mesenchymal progenitors and multipotent/pluripotent lineage stem cells (Berger et al. 2006; Erices et al. 2000). Since these materials are considered to be waste products, UCB cells may be easily procured without damage to donors, thereby circumventing ethical issues. Another important characteristic of UCB cells is that they are more juvenile than those collected from adult tissues; therefore, these cells are easier to expand in culture, more tolerant to human leukocyte antigen (HLA) disparities, and significantly lower risk for immune rejection (Danby and Rocha 2014). The neurological pluripotency of UCB cells has been studied in several reports. Jang et al. showed that cord-derived hematopoietic stem cells could be differentiated into neuronal and glial cells (astrocytes and oligodendrocytes) using retinoic acid (Jang et al. 2004). Similarly, non-hematopoietic stem cells in UCB (most likely mesenchymal progenitors) also process the capability to differentiate into neural-like cells in vitro (Buzanska et al. 2006). Although the pluripotency of UCB toward neuronal lineage is beneficial for transplantation, the limited amount of cells which could be collected remains the main drawback for the utilization of UCB. Because of the restricted volume of cells collected from cord blood, the amount of stem cells in UCB is 10-fold less than that of bone marrow. As a result, UCB cells only are applicable in children or young adults (Moise 2005). Several strategies have been proposed to overcome this problem. For example, transplantations with double unit cord blood and ex vivo expansion of UCB cells have proved to offer better outcomes (Brunstein et al. 2009; Yoshimi et al. 2008). Therefore, UCB cells are still considered as one of potential resources for transplantation.

## Application of stem cells in Parkinson’s disease

### Parkinson’s disease

Parkinson’s disease (PD) is the second most common neurodegenerative disorder, affecting 1 % of the population worldwide after the age of 65. The typical symptoms of PD are bradykinesia, rigidity, and resting tremor (Tanner and Goldman 1996). The main pathological features are extensive loss of dopamine (DA) neurons in the Substantia Nigra pars compacta and the accumulations of cytoplasmic eosinophilic inclusions, Lewy bodies (LB) (Forno 1996). The cause of degenerated nigrostriatal dopaminergic neurons remains largely unknown. Current therapeutic choices for PD patients include levodopa, DA agonists, monoamine oxidase inhibitors, and deep brain stimulation (DBS). Generally, the effectiveness of oral medications begins to wear-off after 5 years (Jankovic 2005). Moreover, these treatments cannot repair the damaged DAstriatal projections; therefore, restorative approaches should be considered in order to improve the therapeutic effect. Since PD patients display selective degeneration of SN DA neurons, cell replacement therapies which can produce functional DA neurons may be a valuable therapeutic approach.

To achieve a successful cell-based therapy in PD, some criteria for cell transplantation are generally suggested (Lindvall and Hagell 2000; Lindvall and Kokaia 2006; Lindvall et al. 2004). (1) The cells should possess the molecular, morphological and electrophysiological properties of DA neurons in substantia nigra; (2) the grafts should be able to reverse the motor deficits of PD; (3) the therapy should enable 100,000 or more DA neurons to survive long term in human putamen; (4) the grafted cells should re-establish a dense terminal network throughout the striatum to functionally integrate into host neural circuitries. Here we review the progress of stem cell therapies and discuss the major problems encountered in PD.

### Graft

The content of graft is the critical issue when performing the transplantation. It is currently unknown whether symptomatic relief would be best achieved by implanting a pure population of DA neurons or a graft containing a portion of glial cells. Several studies support the necessary role of astrocytes for neural differentiation during embryonic development, implying that glial cells are important for fate determination of precursors during implantation (Song et al. 2002). Therefore, mesencephalic tissues containing glial cells were most often used in previous studies. Another key issue in performing grafts for PD treatment is that implanting the most suitable subtype of DA neurons is also critical for the outcome of transplantation. DA rich-ventral mesencephalic grafts contain two types of DA neuron progenitors, including A9 Substantia Nigra neurons and A10 dopamine neurons of the Ventral Tegmental Area (Thompson et al. 2005). Only the A9 subtype DA neurons send innervated axons into the striatum in rats (O’Keeffe et al. 2008; Grealish et al. 2010), suggesting that mesencephalic grafts with more A9 subtype DA neurons would be more beneficial for PD treatment.

In late 1980s, clinicians transplanted human embryonic or fetal ventral mesencephalic tissues into PD patients, but the results were varied. In Madrazo and Lindvall’s open-label trials, PD patients showed improvement of Unified Parkinson’s Disease Rating Scale (UPDRS) after receiving fetal DA neuron graft (Madrazo et al. 1988; Lindvall et al. 1989). However, the results from two double-blind trials funded by the National Institutes of Health (NIH) in the 1990s showed no significant effects (Freed et al. 2001; Olanow et al. 2003). Even more, several side-effects have been shown in PD patients who received these transplantations. The results of these two open-label and double-blind trials raise critical issues regarding ethical considerations, and may enhance controversy which can dissuade the potential use of transplants for PD.

In the following, we will review different alternative sources for the transplantation in PD.

#### ES cell-derived DA neurons

ES cells are one important source that has been used to differentiate into DA neurons in the laboratory. Rodent and human ES cell-derived DA neurons have been shown to survive and function after transplantation into the striatum of PD rats (Kim et al. 2002; Yang et al. 2008). In 2005, Takagi et al. reported that primate ES cell-derived DA neurons survived in the putamen of 1-methyl-4-phenyl-1, 2, 3, 6-tetrahydropyridine (MPTP)-lesioned monkeys. Furthermore, the uptake of [^18^F]-DOPA also increased 14 weeks after transplantation, suggesting exogenous ES cell-derived DA neurons could offer the functional recovery of DA neurons. Until now, ES cells are still the most promising source to differentiate into DA neurons (Kim et al. 2002; Rodriguez-Gomez et al. 2007); however, the efficiency to differentiate into DA neurons and the survival rate of these neurons after transplantation are still low. For example, prior reports showed less than 300 tyrosine hydroxylase (TH)-positive neurons survived after transplanting 100,000–400,000 ES cells into the striatum (Brederlau et al. 2006; Ben-Hur et al. 2004). Therefore a critical issue that must be resolved to enhance recovery after transplantation in PD is improvement of the differentiation and survival rate.

#### iPSC-derived DA neurons

Since the iPSC technique was established in 2006 (Takahashi and Yamanaka 2006), another cell source to generate DA neurons was provided. In 2008, DA neurons were first generated from mouse iPSCs and transplanted into the striatum of a rat PD model, thereby alleviating the symptoms of PD (Wernig et al. 2008). In 2010, DA neurons differentiated from iPSCs of PD patients were transplanted into PD transgenic rats, and these neurons survived for several months and further alleviated the symptoms of PD (Hargus et al. 2010). Most importantly, these transplanted cells did not display α-synuclein positive inclusion bodies in hosts, suggesting that performing autografts in PD patients may be a viable option. However, several reports have mentioned patient-derived iPSCs are still more vulnerable to PD because of mutations or epigenetic markers in cells (Badger et al. 2014; Beevers et al. 2013; Sanchez-Danes et al. 2012). Furthermore, the risk of tumor formation still needs to be minimized before clinical application can be realized (Petit et al. 2014). Since the technique of iPSC production was only developed a few years ago, further optimization may overcome these drawbacks.

#### NSCs and NSC-derived DA neurons

NSCs are multipotent stem cells that are defined as “neurally” destined cells and retain their regional specificity (Horiguchi et al. 2004). Therefore, NSCs that are derived from a primarily DA location, such as ventral mesencephalon (VM), should be the most appropriate source of DA neurons. Several reports have shown the potential ability of NSCs to differentiate into DA neurons (Tan et al. 2014, 2015), and also demonstrated the improvement of symptoms in PD models after transplantation of NSC-derived DA neurons (Parish et al. 2008; Redmond et al. 2007). Additionally, overexpression of different genes, such as Lmx1a, glial cell line-derived neurotrophic factor (GDNF), Brn4 and TH, in NSCs has enhanced the beneficial effects of NSC-derived DA neurons (Tan et al. 2014; Wu et al. 2015; Wakeman et al. 2014). In animal studies, NSC-derived DA neurons overexpressing Nurr1, a critical factor involved in DA specification and survival, has led to functional improvement in rats treated with 6-hydroxydopamine (6-OHDA), a toxic-induced PD model (Park et al. 2006). However, the survival rate of TH positive neurons after transplantation was less than 4.3 % (Park et al. 2006; Studer et al. 1998). In 2008, Parish et al. transfected Wnt5a into NSCs from mouse VM, generating tenfold more DA neurons with TH positive signal than the conventional FGF2-treated NSCs from VM and causing functional recovery in 6-OHDA mice (Parish et al. 2008). Aside from VM, researchers have also derived NSCs from the SVZ, which is another well-known source for these cells. The transplantation of NSCs from SVZ enhanced the recovery of PD symptoms, but the survival rate of these cells was still low (Meissner et al. 2005; Richardson et al. 2005). Therefore, the most important issue that must be overcome in order to achieve NSC transplantation is to increase the cell number and survival rate of transplanted cells.

#### MSCs

Unlike studies using other stem cells, MSCs were grafted into PD models without differentiation in vitro in most studies; however, the spontaneous differentiation ability of BMSCs after transplantation is low (Mezey et al. 2000). Li et al. bilaterally injected BMSCs into striatum of MPTP-lesioned mice, and these cells showed TH immunoreactivity and promoted motor recovery (Li et al. 2001). However, only 0.8 % of implanted cells expressed TH immunoreactivity. To address the low differentiation rate, delivery of Notch1 intracellular domain (NICD), basic fibroblast growth factor (bFGF), forskolin, ciliary neurotrophic factor (CNTF) and GDNF has been used to efficiently induce BMSC differentiation into neuronal cells and increase the proportion of TH-positive cells (Dezawa et al. 2004). Moreover, the transplantation of these treated cells into 6-OHDA rats prevented DA neurons from degeneration (Glavaski-Joksimovic et al. 2009). Most importantly, in a clinical trial using BMSCs treated with bFGF, workers transplanted cells unilaterally into the ventricular zone of advanced PD patients, who showed modest clinical improvement at 12 months and no tumor formation (Venkataramana et al. 2010). This result suggests that BMSCs may be a good choice concerning the issue of safety.

MSCs isolated from umbilical cords have also shown beneficial effects in 6-OHDA PD models (Mathieu et al. 2012; Weiss et al. 2006). However, the low differentiation potential of UCB cells is similar to that of BMSCs. To improve the differentiation rate, UCB cells were cultured with sonic hedgehog and fibroblast growth factor-8 (FGF-8). Cells treated in this manner could reach 12.7 % neuronal differentiation, and these cells successfully ameliorated apomorphine-induced rotations in 6-OHDA lesioned rats (Fu et al. 2006). Overall, transplantation of undifferentiated MSCs or differentiated UCBs could all improve the symptoms of PD. Since there is no report to compare the difference between these cells, further studies will be necessary to conclude which source would be preferable.

In summary, iPSCs, NSCs, and MSCs are the most likely sources of stem cells for PD therapy. In all cases, autografts may be used as demonstrated by several successes in rodent and primate PD models. However, the diverse differentiation methods, low production rate, and low survival rate after transplantation are still obstacles that need to be overcome before clinical use.

### Patient selection

The status of PD patients is a critical factor affecting the outcome after transplantation. Though prior clinical studies have reported diverse effects of transplantation in PD patients (Madrazo et al. 1988; Lindvall et al. 1989; Freed et al. 2001; Olanow et al. 2003), there are some conclusions that may be drawn from detailed analysis. Piccini et al. implanted embryonic ventral mesencephalic tissues into putamen or caudate of nine patients, and [^18^F]-DOPA PET was performed preoperatively and 1 or 2 years post-operatively. According to their results, patients without dopaminergic denervation outside the grafted striatal areas showed the best functional outcome after transplantation (Piccini et al. 2005). In Olanow’s trial, less severe patients (UPDRS <50 points during “off” medication) responded significantly better to fetal grafts (Olanow et al. 2003). Freed and coworkers suggested that patients with younger age or better levodopa response before surgery might benefit more from cell transplantation (Freed et al. 2001). Ma et al. used [^18^F]-DOPA PET to evaluate the outcome of 33 participants 2–4 years after transplantation and also found that younger recipients had better clinical improvement (Ma et al. 2010). In summary, individuals who are young and have better preoperative levodopa responsiveness will be more suitable for cell transplantation. In addition, patients with DA neurons loss restricted to the caudate-putamen will also receive more symptomatic benefit after transplantation.

### Immune response

Although the brain is regarded as an immune-privileged site, the host immune system still responds to the grafts. The interaction between implantation and the endogenous immune system affects the survival of grafted cells. In several clinical trials, transplantation without adequate immunosuppression may have led to poor outcomes (Freed et al. 2001; Olanow et al. 2003), while transplantation with an immunosuppressant, such as cyclosporine, azathioprine and prednisolone, produced better effects (Lindvall et al. 1989). Unfortunately, patient symptoms deteriorated after withdrawal of immunosuppression, and autopsy showed grafts were surrounded by activated microglia and immune reactivity (Olanow et al. 2003). These results imply that immune reactions exert a negative effect during transplantation, and it would be necessary to use an immunosuppressant in combination with grafting; however, further studies are still needed to determine the optimal immunosuppressant and the duration of treatment.

### Major issues after grafts

According to the reports of several cell-based studies in both animals and humans, there are two major concerns related to grafts.

#### Graft Induced Dyskinesia (GID)

The occurrence of dyskinesia after transplantation was first reported by Defer et al. (1996), but did not receive much attention until Freed’s trial in 2001 (Freed et al. 2001). They described the development of “graft induced dyskinesia (GID)” in 15 % of transplanted patients 1 year after transplantation. This unexpected symptom reached 56 % in Olanow’s study (Olanow et al. 2003). It is speculated that the number of grafted cells used in the surgery-controlled clinical trials was less than those of other more successful studies (Lindvall et al. 1989; Freed et al. 2001). Additionally, immunosuppression may be also an important factor since dyskinesia did not develop until immunosuppression withdrawal in several reports (Olanow et al. 2003; Piccini et al. 2005; Lane et al. 2008). The other possible issue is that heterogeneous grafts were found in ventral putamen, containing serotonergic neurons. These grafts led to islands of reinnervation and abnormal production of DA (Ma et al. 2002; Carlsson et al. 2009). Therefore, transplanting sufficient number of cells containing a pure population of DA neurons in basal ganglion with immunosuppression is suggested to avoid the development of GID.

#### Grafts affected by PD process

Evidence that PD pathology may propagate from host to grafts is emerging (Kordower et al. 2008a, b; Li et al. 2008). The presence of LBs and Lewy neurites in grafted DA neurons were generally observed 11–16 years after human fetal mesencephalic transplantation (Kordower et al. 2008a, b; Li et al. 2008; however,α-synuclein staining is generally not detectable in adults younger than 20 year-old (Chu and Kordower 2010). These observations imply that PD pathology can be transferred from host to graft (Visanji et al. 2013). The exact reasons for this negative outcome remain unresolved. A recent report showed that fetal cell transplantation in two PD patients remains highly functional even 15–18 years after surgery (Kefalopoulou et al. 2014). Therefore, although the spread of PD pathology may occur after transplantation, the period of beneficial effects from transplantation is still longer than that of current medications, suggesting stem cells still could be a potential clinical therapy.

### Mechanisms of stem cell therapy in PD

From the positive results of prior studies, the effects of stem cell therapy on PD can be classified into two categories. The first is a direct repair pathway, which includes augmenting endogenous neurogenesis, DA neuron differentiation (Park et al. 2012), DA release (Rodriguez-Gomez et al. 2007; Bouchez et al. 2008), striatum reinnervation (Kordower et al. 1995) and neural circuits integration (Piccini et al. 2000; Bjorklund et al. 2002). The second is indirect repair system through trophic factors. Stem cells express various neurotrophic factors, such as brain derived neurotrophic factor (BDNF), nerve growth factor (NGF), cerebral dopamine neurotrophic factor (CDNF) or glial-derived neurotrophic factor (GDNF), and facilitate DA neuronal differentiation and maintenance. These bystander effects are especially likely to result from grafts comprised of NSCs and MSCs (Rafuse et al. 2005; Tolar et al. 2010; Yasuhara et al. 2006; Lu et al. 2003). However, it is still hard to distinguish clearly which pathway plays a dominant role, and, as a result, it is generally assumed that both direct and indirect pathways contribute to the beneficial effects after transplantation.

## Conclusion

There is still no cure for PD since the precise mechanisms of this disease are largely unknown. High expectations have been placed on stem cell therapy to achieve this goal since many of the cell-based studies on PD animal models have shown positive results; however, the outcomes in clinical trials have not been consistent or convincing. This is possibly due to a combination of factors, such as patient selection, amount and mode of tissue engraftment and the level of immunosuppression. Additionally, another side effect to be considered is GID. Fortunately, grafted tissues were not affected by PD progression within 10 years after transplantation, so the treatment of PD with stem cell grafts is still a promising direction. The major advantage of this strategy is the restorative and trophic abilities of the grafted cells which reach far beyond drugs prescribed in current practice.
